# Ventricular Fibrillation Recurrences in Successfully Shocked Out-of-Hospital Cardiac Arrests

**DOI:** 10.3390/medicina57040358

**Published:** 2021-04-07

**Authors:** Daniela Aschieri, Federico Guerra, Valentina Pelizzoni, Enrico Paolini, Giulia Stronati, Luca Moderato, Giulia Losi, Paolo Compagnucci, Michela Coccia, Michela Casella, Antonio Dello Russo, Gust H. Bardy, Alessandro Capucci

**Affiliations:** 1 Cardiology Department, Civil Hospital, 29015 Castel San Giovanni, Italy; dani.aschieri@gmail.com (D.A.); giulia.losi09@gmail.com (G.L.); michelagiovannacoccia1990@gmail.com (M.C.); 2 Department of Biomedical Science and Public Health, Cardiology and Arrhythmology Clinic, University Hospital “Ospedali Riuniti Umberto I-Lancisi-Salesi”, Marche Polytechnic University, 60020 Ancona, Italy; g.stronati@staff.univpm.it (G.S.); paolocompagnucci1@gmail.com (P.C.); a.dellorusso@staff.unipvm.it (A.D.R.); profacapucci@gmail.com (A.C.); 3 Cardiology Department, “Guglielmo da Saliceto” Hospital, 29121 Piacenza, Italy; valepelli@libero.it (V.P.); moderatoluca@gmail.com (L.M.); 4 Cardiology Department, “Ospedali Riuniti Marche Nord”, 61121 Pesaro, Italy; enripaolini1985@gmail.com; 5 Department of Clinical, Special and Dental Sciences, Cardiology and Arrhythmology Clinic, University Hospital “Ospedali Riuniti Umberto I-Lancisi-Salesi”, Marche Polytechnic University, 60020 Ancona, Italy; m.casella@staff.univpm.it; 6 Seattle Institute for Cardiac Research, Seattle, WA 98195, USA; gbardy@sicr.org

**Keywords:** cardiac arrest, cardiopulmonary resuscitation, chest compressions, sudden cardiac death, ventricular fibrillation, defibrillation

## Abstract

*Background and Objectives:* The prognostic impact of ventricular fibrillation (VF) recurrences after a successful shock in out-of-hospital cardiac arrest (OOHCA) is still poorly understood, and some evidence suggests a potential pro-arrhythmic effect of chest compressions in this setting. In the present analysis, we looked at the short-term and long-term prognosis of VF recurrences in OOHCA. And their potential association with chest compressions. *Materials and Methods:* The Progetto Vita, prospectively collecting data on all resuscitation efforts in the Piacenza province (Italy), was used for the present analysis. From the 461 OOHCAs found in a shockable rhythm, only those with optimal ECG tracings and good audio recordings (160) were assessed. Rhythms other than VF post-shock were analyzed five seconds after shock delivery and survival to hospital admission, hospital discharge, and long-term survival data over a 14-year follow-up were collected. *Results:* Population mean age was 64.4 ± 16.9 years, and 31.9% of all patients were female. Mean time to EMS arrival was 5.9 ± 4.5 min. Short- and long-term survival without neurological impairment were higher in patients without VF recurrence when compared to patients with VF recurrence, independently from the pre-induction rhythm (*p* < 0.001). After shock delivery, VF recurrence was higher when chest compressions were resumed early after discharge and more vigorously. *Conclusions:* VF recurrences after a shock could worsen short and long-term survival. The potential pro-arrhythmic effect of chest compressions should be factored in when considering the real risks and benefits of this procedure.

## 1. Introduction

Cardiopulmonary resuscitation (CPR) is commonly perceived as being beneficial in out-of-hospital cardiac arrest (OOHCA), and the current guidelines recommend prompt CPR immediately after defibrillation [1]. Simultaneously, it is well known that a direct, blunt force applied to the chest could also induce ventricular fibrillation and sudden death, in an event called “commotio cordis” [2]. The blow described as the optimal trigger for commotio cordis shares many characteristics with the guideline-advised technique for chest compressions, [3] which could therefore potentially increase the incidence of ventricular fibrillation (VF) recurrence after the first effective shock. The incidence of recurrent ventricular fibrillation (VF) varies from 18% to 72% [4,5], and the number of recurrent VF is negatively associated with survival to admission [4].

In the present analysis of the Progetto Vita experience (www.progetto-vita.eu, accessed on 18 March 2021), we examine survival in a population of OOHCA victims in Piacenza, Italy who were found with a shockable rhythm and promptly defibrillated. We tested the hypothesis that VF recurrence could be associated with a long-term survival in OOHCA patients found in a shockable rhythm. We also tested the hypothesis that chest compressions may be associated with VF recurrence, particularly when they are performed vigorously and promptly after a successful shock.

## 2. Materials and Methods

The aim and methodology of Progetto Vita have been previously reported [6,7]. In brief, all OOHCAs occurring in the city of Piacenza and nearby county were documented according to standard reporting criteria. Transient losses of consciousness, including syncope, were excluded from the analysis, as were cardiac arrests following administration of cardioactive medications during an acute myocardial infarction. Survival to hospital admission, survival to hospital discharge, and long-term survival over a 14-year follow-up was prospectively assessed. Patient vital status up until 1 August 2017, was validated for 100% of OOHCA victims via the Italian national registry of death and review of medical records.

For the present analysis, the AED tracings of all consecutive patients enrolled from 2001 to 2014 into the Progetto Vita registry and presenting with a shockable rhythm were prospectively collected. All analyses were obtained by consensus of two independent investigators experienced in analysis of cardiac rhythms (E.P. and F.G.). ECGs were excluded from the study if tracings were incomplete or displayed sub-optimal quality, not allowing a clear detection of cardiac rhythm or chest compression-related artifacts.

**Figure 1:**
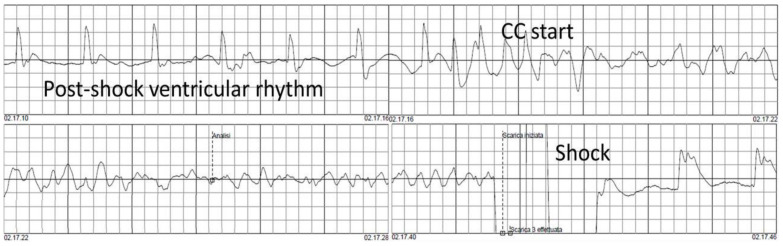
VF recurrence during chest compressions.

**Figure 2:**
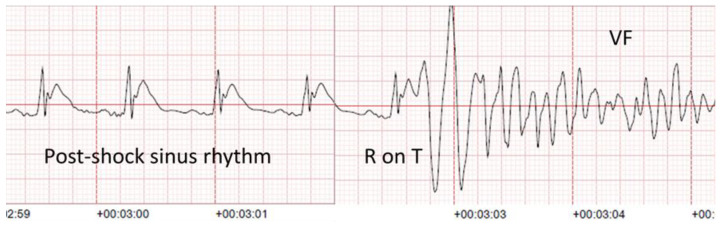
Spontaneous VF recurrence due to “R on T” phenomenon.

Rhythms other than VF post-shock were analyzed five seconds after shock delivery and were classified as asystole or spontaneous electrical activity (SEA). As the simultaneous presence of a pulse was obviously unknown during any rhythm other than VF or asystole, SEA included all cardiac rhythms, whether perfusing or pulseless electrical activity (PEA). Chest compressions were defined by ECG compression artifacts with audio voice confirmation, as is the standard practice in Piacenza. Figure 1 shows an example of a VF recurrence occurred during the chest compression artifact. Figure 2 shows a spontaneous VF recurrence unrelated to chest compressions.

### Statistical Analysis

Our primary endpoint was to test the association between VF recurrence and long-term survival in OOHCA patients found in a shockable rhythm. Our secondary endpoint was to test the association between chest compressions and VF recurrence.

Continuous variables were described as mean ± standard deviation, or median and first-to-third quartile range if not normally distributed. Categorical variables were described as absolute values and percentages. The χ2 test was used to compare rVF incidence and chest compression-related variables, as well as survival to hospital admission and to hospital discharge between each group. Kaplan–Meier survival curves were used in order to compare survival over the 13-year follow-up. A two-tailed *p*-value ≤ 0.05 was considered as statistically significant. SPSS 21.0 for Windows (SPSS Inc. Chicago, IL, USA) was used for all the statistical analyses.

## 3. Results

### 3.1. General Characteristics

From 1 June 2001 to 1 August 2014, 461 (13.7%) out of 3366 confirmed OOHCAs presented with a shockable rhythm. Of those, 163 (35.4%) had a OOHCA with a good-quality tracing available and were included in the present analysis. To better quantify the potential selection bias due to the high attrition rate, Table 1 reports the general characteristics of the total sample, along with the characteristics of the excluded and included patients. Mean age was 64.4 ± 16.9 years, and 52 (31.9%) of all patients were female. Mean time to first EMS team arrival (either BLS or ALS crew) was 5.9 ± 4.5 min, with 37 (22.7%) OOCHAs occurring in public places and 126 (77.3%) at home. In 14 cases (8.6%) Progetto Vita was the first responder, while in the other 149 cases (91.4%) EMS arrived on the site first. After the Progetto Vita first responder was defibrillated, EMS took over patient management, including the use of chest compressions.

Out of all 163 OOHCAs with a first shockable rhythm, 109 (66.9%) survived to hospital admission and 64 (39.3%) survived to hospital discharge with no neurological impairment.

### 3.2. VF Recurrence and Survival

**Figure 3:**
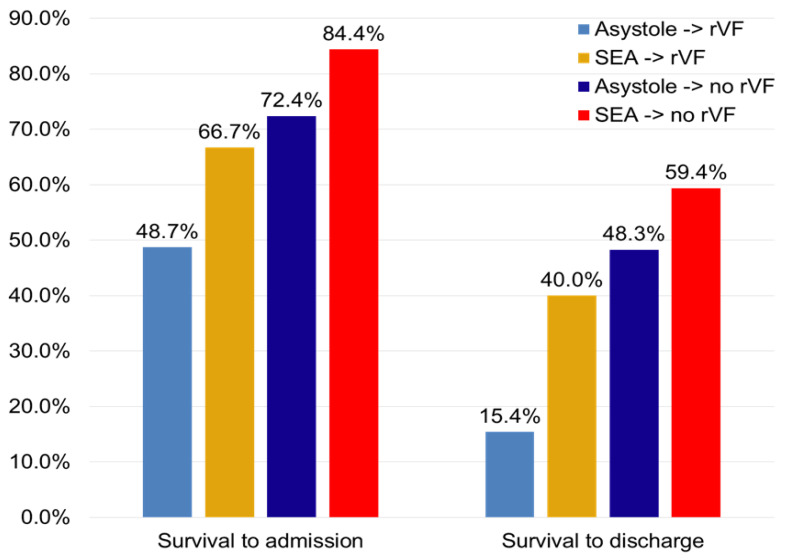
Short-term survival according to post-shock rhythm and rVF. rVF: recurrent VF; SEA: spontaneous electrical activity.

**Figure 4:**
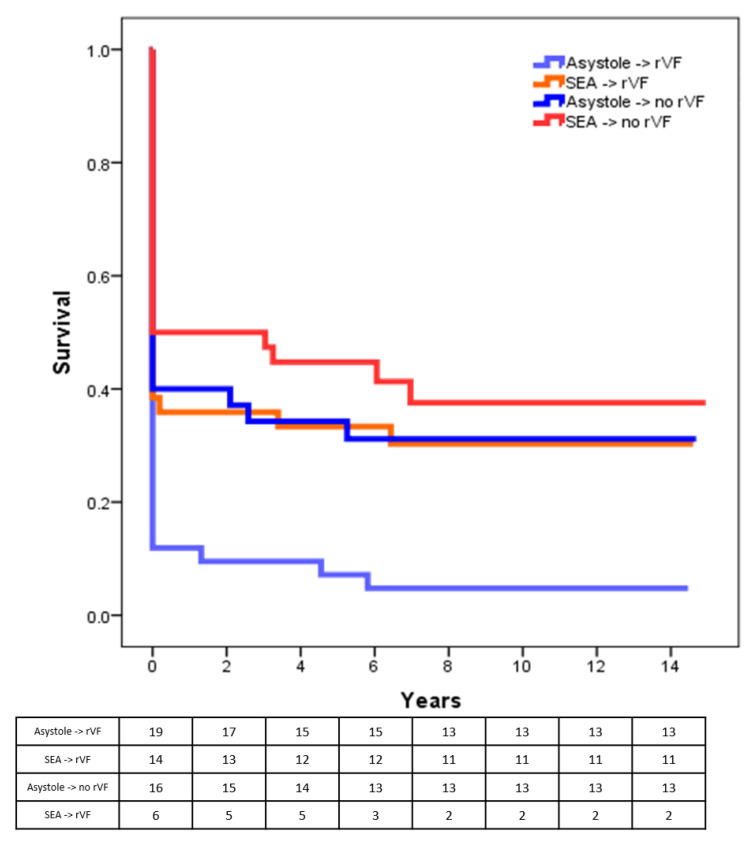
Long-term survival according to post-shock rhythm and rVF. rVF: recurrent VF; SEA: spontaneous electrical activity.

The 163 patients were divided into four groups, according to the post-shock rhythm and the incidence of VF recurrence. Mean time to first EMS team arrival was similar between patients with and without VF recurrence (5.8 ± 3.2 vs. 6.0 ± 3.9 min; *p* = 0.772). Overall, asystole was the post-shock rhythm in 82 patients (50.3%), of which 46 (28.2%) experienced at least one recurrence. The other 81 (49.7%) showed an SEA, which later degenerated into another VF in 43 patients (23.3%). Short-term and long-term survival for each group is shown in Figure 3 and Figure 4, respectively.

### 3.3. After-Shock Chest Compressions

**Figure 5:**
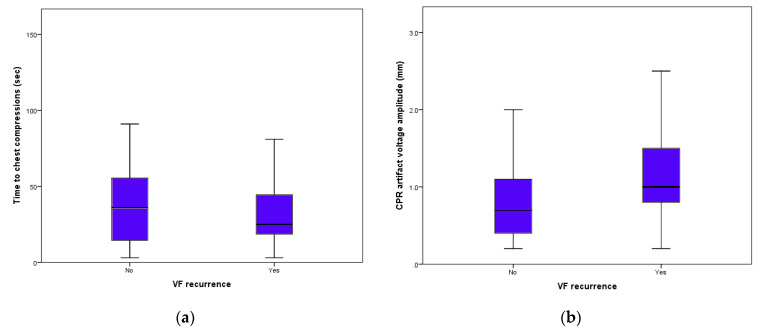
Time from shock to start of chest compressions (**a**) and chest-compression artifacts height (**b**) according to the presence or absence of VF re-induction.

Overall, chest compressions were performed 241 total times after a VF was successfully terminated by a shock. Median time from shock to chest compression start was 19 *s* (1st–3rd quartile range 6 to 41 s) and the median voltage of the CPR artifact was 1.0 mV (1st–3rd quartile range 0.6 to 1.5 mV). Early chest compressions, defined as a CPR start within 19 s, was associated with a higher incidence of rVF (66.7% vs. 43.5%; *p* < 0.001). Vigorous chest compressions, defined as a CPR artifact ≥ 1 mV, was also associated with a higher VF recurrence incidence (79.2% vs. 42.7%; *p* < 0.001). Figure 5 shows the scatter boxes of time to chest compression start and CPR artifact voltage according to presence or absence of VF recurrence. Post-shock CPR was performed in 56 (87.5%) patients surviving hospital discharge with no neurological impairment and in 89 (89.8%) patients that either died or survived with neurological impairment (*p* = 0.63).

## 4. Discussion

The observations stemming from the present study are no doubt controversial. In the present analysis of the Progetto Vita experience, we documented an increased incidence of both VF recurrence and subsequent mortality when CPR was performed after a first appropriate shock. While our data suggest that prompt and vigorous chest compressions after the first shock was associated with re-induced VFs, the observational nature of our study does not allow implying a cause-effect relationship between CC and VF re-induction. Moreover, while it has already been documented that CPR can cause serious side effects such as coronary embolism, great vessel lacerations and organ rupture [8,9], its potential pro-arrhythmic effect is neither generally considered or understood.

On the one hand, our findings confirm the ones from Berdowski and colleagues [5], who underlined a temporal relationship between the start of chest compressions and VF recurrence onset. Using impedance signals in order to detect CPR starting times, they demonstrated that the earlier the rescue team started CPR, the earlier the VF reoccurred. On the other hand, Conover et al. showed that the risk of VF recurrence was the same whether chest compressions were resumed immediately or 30 s after a shock, and found no relationship between VF re-induction and age, sex, or time to arrival [10]. While the two studies differ in terms of guidelines adopted, statistical methods, and patients enrolled, still they provided conflicting evidence and neither focused on long-term mortality associated with CPR and recurrent VF. Our data, while supporting a reasonable hypothesis, nevertheless did not prove conclusive in settling the issue. This was also due to the lack of an impedance signal, that could probably have increased the ECG reviewers’ sensitivity and sensibility in detecting CPR start and stop. Moreover, only 35% (163 out of 461) of all available tracings were included in the analysis due to sub-optimal quality. While the patients whose tracing were excluded had similar characteristics to the ones analyzed (Table 1), still it is possible that the tracing selection could represent a bias.

The pathophysiologic explanation between chest compressions and VF reinduction could not be as straightforward as previously thought. Similarly to how low-energy, blunt hits to the thoracic wall can produce VF in commotio cordis in association with long-short activation sequences during the vulnerable period, the repetitive impact of CPR could produce stretch-mediated or cardiac tear or coronary crush mediated electrophysiological changes in patients with a spontaneous rhythm [11].

Application of a direct force to the chest can exert delayed afterdepolarization-like effects through mechano-electric feedback mediated by mechanosensitive ion channels [12]. These channels have been described in the heart tissue and are defined by status in response to a mechanical stimulus, translating kinetic energy into chemical signals. Osorio and colleagues demonstrated that chest compressions can produce intermittent ventricular capture in a swine model [11] and in humans [13]. While this phenomenon could produce positive results during asystole, still some of the patients studied experienced VF during chest compressions or shortly following their initiation [13]. In this setting, a mechanical stretch during diastole can lead to delayed afterdepolarization, which may reach the action potential threshold and produce a triggering premature beat. Therefore, stronger chest impacts and an early start of chest compressions on a recently shocked myocardium could be more likely to provoke an action potential and re-induce VF, as appears to be the case from our data. There is also supporting evidence for this possibility from a prospective registry of in-hospital CA in Norway [14] that showed that CPR during the first three minutes was negatively associated with survival, and also debated whether performing two minutes of CPR after a shock could actually be harmful, suggesting repeated shocks with escalating energy as a better alternative in cases of VF persistence than initiation of CPR [15]. Surely, while the aforementioned mechanisms could explain VF recurrences during SEA, they still fall short of explaining outcomes when asystole is the post-shock rhythm and ventricular “capture” from CPR does not occur. In these cases, coronary reperfusion during chest compressions could play a role. Reperfusion arrhythmias were originally described after restoration of coronary circulation in acute coronary syndromes [16] and could present as pulseless ventricular tachycardia or ventricular fibrillation [17]. Coronary perfusion pressures, myocardial blood flow, and oxygen delivery are known to differ widely from the beginning to the end of each compression cycle [18]. Consequently, it is feasible to hypothesize that these variations could alter membrane excitability and provoke transient depolarization and electrical uncoupling as seen in animal ischemia models, [19] thus promoting VF recurrence.

Regarding VF recurrence as a marker of mortality, recent papers show that VF re-occurrence and time in recurrent VF is a marker of worse survival in OOHCA treated by first responders as well as ALS [20,21]. This notion holds true even in patients with implantable cardioverter-defibrillators, in whom clusters of ventricular arrhythmias are called an “electrical storm” and are associated with a 3-fold risk of death [22,23]. Our data showed that VF recurrence could be considered a mortality marker in OOHCA presenting with a shockable rhythm, despite the rhythm attained after the first shock. In this setting, experiencing a second VF after the first one is associated with a reduction of survival to discharge of 32.9% in patients with post-shock asystole and of 19.4% in patients with a post-shock organized rhythm, and with a parallel decrease in long-term survival.

Another alternative explanation is that there is worse survival associated with rVF because of epinephrine intoxication [24], or rVF is merely a marker of sicker patients and impaired myocardium [25], where mitochondrial function is decreased, cardiac ATP production is slowed, and reactive oxygen species are overproduced [26]. Yet, one would think that the CPR effort would improve such problems of an impaired myocardium. A minor role could be played by the performance of the EMS team members, which could see their efforts thwarted by the recurrence of VF. However, we were not able to test this hypothesis, which has currently no supporting evidence, as socioemotional stress seems to have no effect on the resuscitation performance of healthcare personnel [27].

## 5. Conclusions

While our data are observational in nature and do not prove a causal relationship between CPR and VF recurrence, the potential pro-arrhythmic effect of chest compressions should be considered when considering the real risks and benefits of this procedure. That said, additional evidence could be collected first in a pig model of persistent VF, and time of initiation of CPR being progressively longer.

Like many presumed beneficial procedures, CPR stands alone in the fact that it has never been tested prospectively in a controlled, randomized manner looking at long-term mortality. Although “doing nothing” may seem antithetical to responding to someone in cardiac arrest, this would not be the first time in medical history that restraint from a procedure proves beneficial in some circumstances. We conclude that VF recurrence after a first successful shock appears to worsen long-term survival independently from the pre-induction rhythm and clinical measures of post-arrest survival.

## Figures and Tables

**Table 1:** General characteristics of all patients presenting with a shockable rhythm, divided according to the availability of good-quality tracing.

	All Shockable Rhythms	Excluded Patients	Included Patients
	461	298	163
Age (years)	63.3 ± 15.4	62.6 ± 16.3	64.4 ± 16.9
male gender	325 (70.5%)	214 (71.8%)	111 (68.1%)
mean time to arrival (min)	5.8 ± 3.7	5.7 ± 4.2	5.9 ± 4.5
**Location of cardiac arrest**			
Home	360 (78.1%)	234 (78.5%)	126 (77.3%)
Public places	101 (21.9%)	64 (21.5%)	37 (22.7%)
**Initial responder**			
Progetto Vita	65 (14.1%)	40 (13.4%)	25 (15.3%)
BLS EMS	219 (47.5%)	133 (44.6%)	86 (52.8%)
ACLS EMS	177 (38.4%)	125 (41.9%)	52 (31.9%)

## Data Availability

Data will be made available upon request to the corresponding author.
